# *Plasmodium malariae* infections as a cause of febrile disease in an area of high *Plasmodium falciparum* transmission intensity in Eastern Uganda

**DOI:** 10.1186/s12936-021-03962-1

**Published:** 2021-10-29

**Authors:** Daniel Ayo, Bakar Odongo, Joseph Omara, Chiara Andolina, Ole Mulder, Sarah G. Staedke, Teun Bousema

**Affiliations:** 1 Infectious Diseases Research Collaboration, Kampala, Uganda; 2 Department of Medical Microbiology, Radboud University Nijmegen Medical Centre, Nijmegen, The Netherlands; 3 Department of Clinical Research, London School of Hygiene and Tropical Medicine, London, UK; 4 Department of Immunology and Infection, London School of Hygiene and Tropical Medicine, London, UK

**Keywords:** *Plasmodium malariae*, *P. falciparum*, Diagnosis, Misdiagnosis, Schizonts, Microscopy

## Abstract

**Background:**

*Plasmodium falciparum* is responsible for the vast majority of (severe) clinical malaria cases in most African settings. Other *Plasmodium* species often go undiagnosed but may still have clinical consequences.

**Case presentation:**

Here, five cases of *Plasmodium malariae* infections from Eastern Uganda (aged 2–39 years) are presented. These infections were all initially mistaken for *P. falciparum,* but *Plasmodium* schizonts (up to 2080/µL) were identified by microscopy. Clinical signs included history of fever and mild anaemia.

**Conclusion:**

These findings highlight the importance of considering non-falciparum species as the cause of clinical malaria. In areas of intense *P. falciparum* transmission, where rapid diagnostic tests that detect only *P. falciparum* antigens are commonly used, non-falciparum malaria cases may be missed.

## Background

In 2019, an estimated 229 million cases and 409,000 deaths due to malaria were reported globally [1]. Most of these occurred in sub-Saharan Africa, and were caused by *Plasmodium falciparum.* Thus, it is not surprising that *P. falciparum* remains the focus of malaria control programmes and most malaria research in Africa. Other *Plasmodium* species are endemic in sub-Saharan Africa, including *Plasmodium ovale, Plasmodium vivax* and *Plasmodium malariae* [2]. These non-falciparum species also cause clinical symptoms, and lead sometimes to severe consequences. Chronic infections with *P. malariae* may last for years, and can cause severe complications in approximately 3% of cases, including nephrotic syndrome, splenomegaly and anaemia [3, 4]. Moreover, *P. malariae* infections have been observed following treatment of *P. falciparum* infections with artemisinin-based combination therapy (ACT) [5, 6].

Different *Plasmodium* species can be differentiated by microscopy but this requires laboratory infrastructure and technical expertise. Infections with *P. malariae* are typically characterized by low parasitaemia [7] and the presence of rosette schizonts (daisy heads) and other parasite life-stages in the peripheral blood [8]. *Plasmodium malariae* is often missed or misdiagnosed by microscopy [9, 10]. However, polymerase chain reaction (PCR) suggests a non-negligible prevalence of *P. malariae* infection, either alone or mixed with other species, in population surveys in African countries, where prevalence of up to 12% has been reported [11, 12].

Rapid diagnostic tests (RDTs), which are simple to use and require minimal training, are increasingly being used to diagnose malaria. In Africa, most RDTs detect histidine-rich protein 2 (HRP2), an antigen that is specific for *P. falciparum;* use of these HRP2 RDTs is likely to contribute further to under-diagnosis of *P. malariae* infections.

Here, five cases are presented of symptomatic, uncomplicated *P. malariae*. These cases were coming from an area of intense *P. falciparum* transmission in Eastern Uganda and were initially misdiagnosed as *P. falciparum* by microscopy.

## Case presentation

Patients described in this case report presented at either Masafu General Hospital or Nagongera Health Centre IV. Masafu General Hospital is a district referral facility in Busia District, an area in south-eastern Uganda that borders Kenya and Lake Victoria, where transmission of *P. falciparum* is perennial and intense [13]. Nagongera Health Centre IV in located in Nagongera sub-county in Tororo district. Nagongera sub-county is predominantly a rural setting, with traditionally very high malaria transmission [14]. Between 2019 and 2020 the total Outpatient Department treatment (OPD) attendance in Masafu General Hospital was 40,471, whilst in Nagongera Health Centre IV 18,624 [15].

In both settings, hospital and health centre, malaria diagnosis is routinely performed by HRP-2 based rapid diagnostic test (Standard Q Malaria Pf Ag, SD Biosensor, South Korea). However, as part of a research study on *P. falciparum*, clinic technicians all received formal microscopy training and thick and thin blood films were used for malaria diagnosis, instead of RDTs. Haemoglobin measurements were performed by Hemocue™ (haemoglobinometer, Angelholm, Sweden).

### Case-patient 1

On January 30th 2020, a 15-year-old male presented to the OPD with history of fever, headache and joint pain for 3 days with no evidence of severe malaria or danger signs. At initial presentation, he had an axillary temperature of 36.8 °C and his physical examination was within normal limits. Malaria parasites were reported on the thick and thin blood film. By thick blood smear, total parasite count was 896 parasites/μL; malaria schizonts were observed at 176 parasites/μL (and included in the total parasite density estimate). Haemoglobin concentration was 12.3 g/dL. The patient was diagnosed with P. falciparum malaria.

### Case-patient 2

On February 19th 2020, a 20-year-old female presented to the OPD with history of fever for 3 days and headache, weakness and joint pain for 2 days. Her temperature on admission was 37.2 °C and her physical examination did not show abnormalities. After reading the thick blood film, malaria infection was diagnosed with a total of 2288 parasites/μL (2080 schizonts/μL). Mild anaemia was detected with haemoglobin of 10.8 g/dL. The patient was diagnosed with *P. falciparum* malaria.

### Case-patient 3

On February 27th 2020, a 39-year-old female presented to the OPD with history of fever and joint pain for 2 days and headache, weakness and low back pain for 4 days. Her axillary temperature on admission was 37.0 °C and she was diagnosed with uncomplicated *P. falciparum* malaria based on a thick blood film with 336 parasites/μL (272 schizonts/μL). Mild anaemia was detected with haemoglobin of 9.5 g/dL.

### Case-patient 4

On March 3rd 2020, a 2-year-old boy presented to the outpatient department with persistent vomiting, anorexia and headache. He had an axillary temperature of 38.3 °C. Microscopy slides were interpreted as *P. falciparum/P. malariae* mixed infection based on presence of 32 ring-stage parasites/μL and 520 schizonts/μL for *P. falciparum* and one trophozoite “band form” for *P. malariae*. Haemoglobin was 7.8 g/dL. The patient was diagnosed with uncomplicated mixed species malaria infection.

### Case-patient 5

On April 29th 2020, a 14-year-old male with mild symptoms of fever, headache, joint pain and anorexia presented to Nagongera Health Center IV, Tororo district. He had an axillary temperature of 37.4 °C. Malaria was diagnosed by microscopy with 4016 parasites/μL (96 schizonts/μL). Haemoglobin was 11.1 g/dL. The patient was diagnosed with uncomplicated *P. falciparum* malaria.

**Fig. 1:**
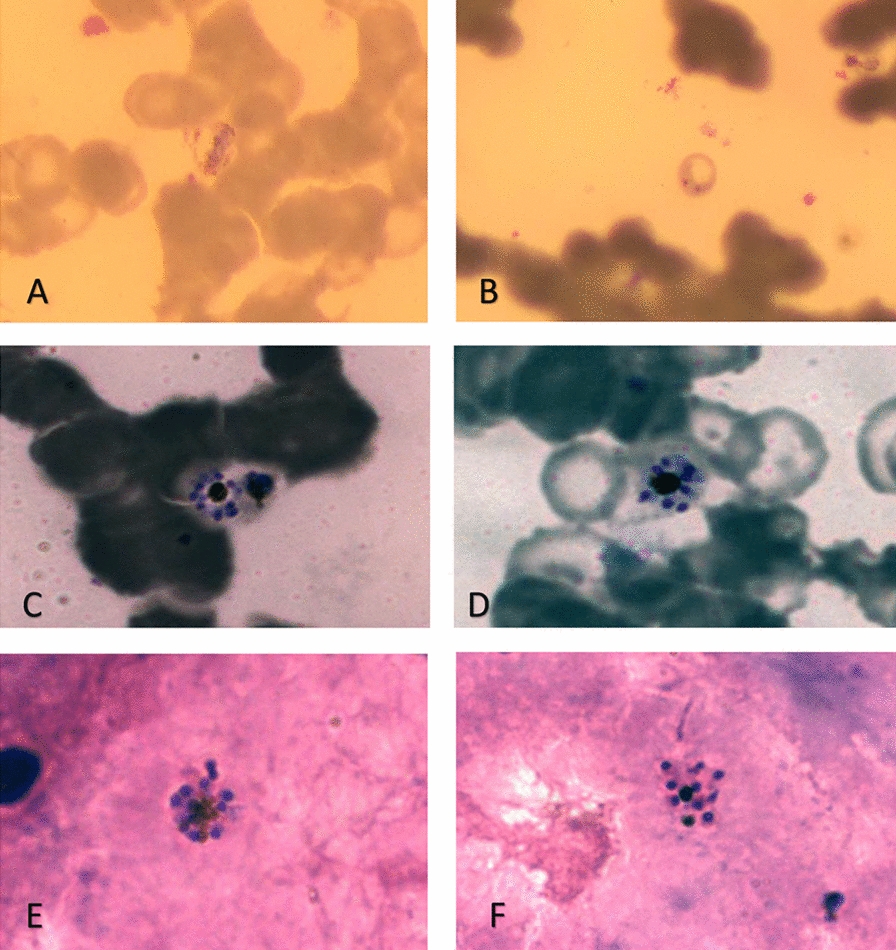
Features of *Plasmodium malariae* parasites in thin and thick blood smears. **A** typical band form mature trophozoite. × 10 ocular, × 100 magnification, thin smear. **B** young trophozoite. Pictures **A**, **B** taken with a smart phone camera. **C**, **D**: “daisy shape” schizonts. × 10 ocular, × 100 magnification, thin film. **E**, **F**: schizonts. × 10 ocular, × 100 magnification, thick smear. **C**–**F**: Pictures taken with Axio Cam MRc-5, Zeiss, Germany. Blood smears were stained with 10% Giemsa solution

All five cases were treated for uncomplicated *P. falciparum* infection according to national Ugandan treatment guidelines with artemether lumefantrine 20 mg/120 mg twice per day for 3 days and discharged home [16]. Although there are some reports of persisting *P. malariae* parasites after artemether-lumefantrine [5, 17], suggestive of reduced treatment efficacy for this species, most available data support the use of artemether-lumefantrine for *P. malariae* [18, 19]. Thick and thin smears were re-read by expert microscopists and representative images taken (Fig. 1). Parasite DNA was extracted from fixed Giemsa-stained thick and thin smears using a modified protocol for isolating genomic DNA from dried blood spots (QIAamp^®^DNA Micro kit, cat numb. 56304) [20]. *Plasmodium* speciation was performed using modified methods described by Snounou et al. [21]. *Plasmodium malariae* parasite DNA was detected in material from all slides; *P. falciparum* was also detected for case 4 (Table 1).

**Table 1:** Patient characteristics at presentation

Case	Age	Gender	Temperature (°C)	Symptoms	Haemoglobin (g/dL)	Trophozoite (rings) count^a^	Schizont count^a^	Diagnosis by microscopy	Diagnosis by 18S qPCR
1	15	Male	36.8	Headache 3d, joint pain 2d, fever	12.3	720	176	Pf	Pm
2	20	Female	37.2	Headache 2d, general body weakness and joint pain 3d, fever 3d	10.8	208	2080	Pf	Pm
3	39	Female	37.0	Fever and joint pain 2d, general body weakness, headache, backache 4d	9.5	64	272	Pf	Pm
4	2	Male	38.3	Persistent vomiting, anorexia, fever, headache	7.8	32	520	Pf + Pm	Pm/Pf
5	14	Male	37.4	Headache, fever 3d, joint pain, anorexia	11.1	3920	96	Pf	Pm

^a^Density per microlitre of blood

## Discussion and conclusions

In these five symptomatic malaria cases, *P. malariae* was the most likely cause of the clinical symptoms. All cases were classified as uncomplicated malaria, although they presented with anaemia, a condition that is often associated with chronic infection with *P. malariae* [3, 4]. In four cases, *P. falciparum* infection was initially diagnosed. Only after an expert microscopist reviewed the blood slides was *P. malariae* identified as the infecting species. The presence of symptomatic *P. malariae* mono-infections has implications for the use of HRP-2 RDTs. In both of the study settings, HRP-2 RDTs are routinely used to diagnose malaria. Since non-falciparum *Plasmodium* species do not express HRP-2 antigen, at least four of these cases would presumably have been missed in routine practice and would not have received malaria treatment.

he cases presented here all reported to the health facilities within a relatively short 13-week period. No effort is made to systematically investigate for non-falciparum malaria among individuals presenting with suspected malaria under standard care. This suggests that a considerable number of patients may present with uncomplicated non-falciparum malaria in this setting. The *P. malariae* cases reported here were older than typical *P. falciparum* cases in the area [22], which could be the consequence of a lower force of infection of non-falciparum malaria, older age at first infection, and higher average age at clinical presentation. However, other studies found *P. malariae* infections predominantly in children [23, 24], further highlighting the need for research into the clinical burden of non-falciparum malaria in African settings. Identifying the species of *Plasmodium* infections using molecular diagnostic techniques is needed to quantify the burden of malaria in areas where multiple species may be present.

## Data Availability

Not applicable. All data are included in the manuscript.

## Abbreviations

HRP2: Histidine-rich protein 2
OPD: Outpatient department treatment
PCR: Polymerase chain reaction
RDTs: Rapid diagnostic tests
